# Supplementary material for: Integrated Analysis and Identification of Critical RNA-Binding Proteins in Bladder Cancer
Source: Cancers (Basel). 2022 Jul 31;14(15):3739. doi: 10.3390/cancers14153739 (PMC9367304; doi:10.3390/cancers14153739)
Supplement: Supplementary file 1 [file cancers-14-03739-s001.zip › cancers-1796439-supplementary.pdf]

Supplementary Materials

Integrated Analysis and Identification of Critical RNA-Binding Proteins in Bladder Cancer

Lijiang Gu, Yuhang Chen, Xing Li, Yibo Mei, Jinlai Zhou, Jianbin Ma, Mengzhao Zhang, Tao Hou, Dalin He and Jin Zeng

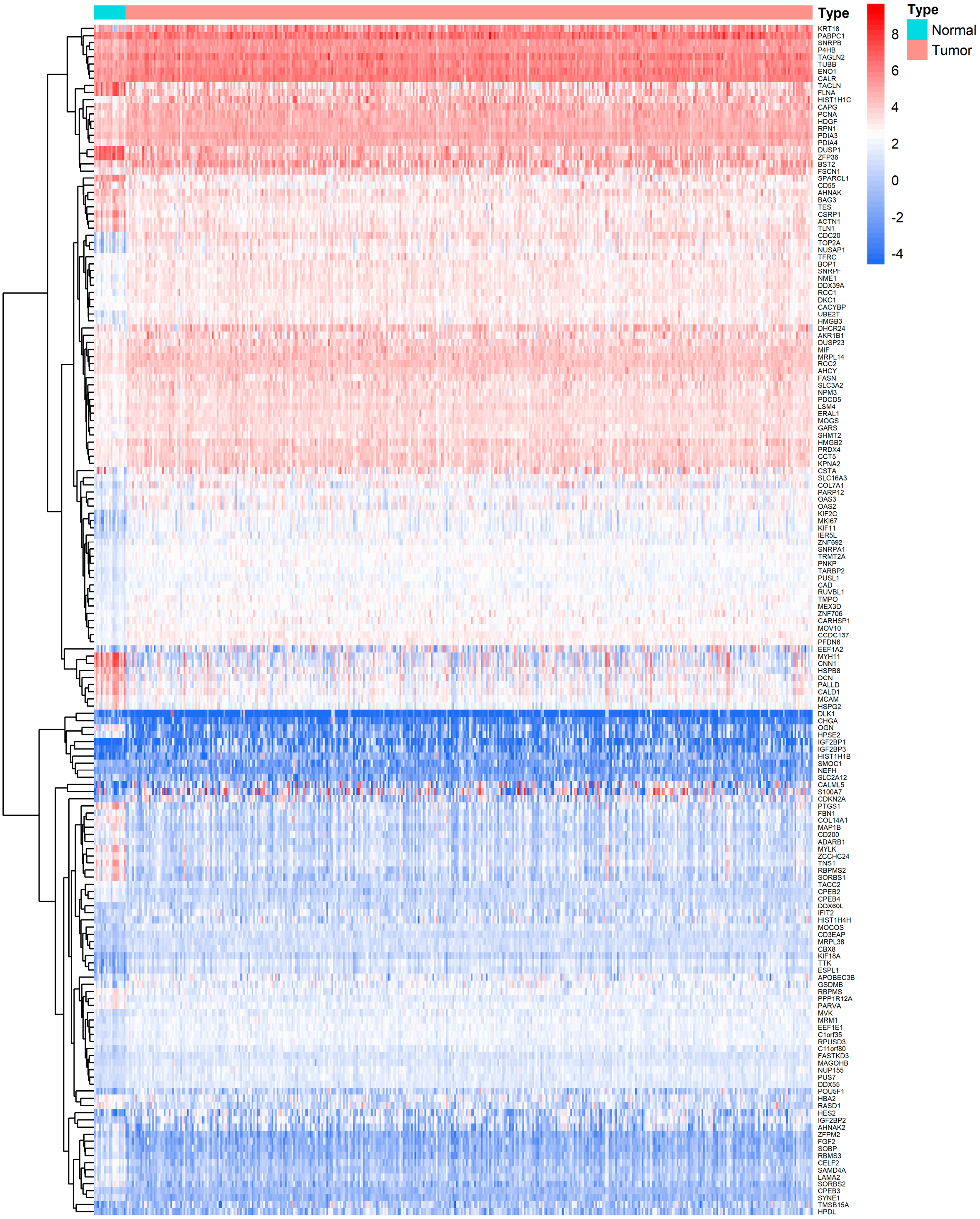

Figure S1. Heat map of differentially expressed RNA-binding proteins (RBPs) in bladder cancer (BC) from TCGA.

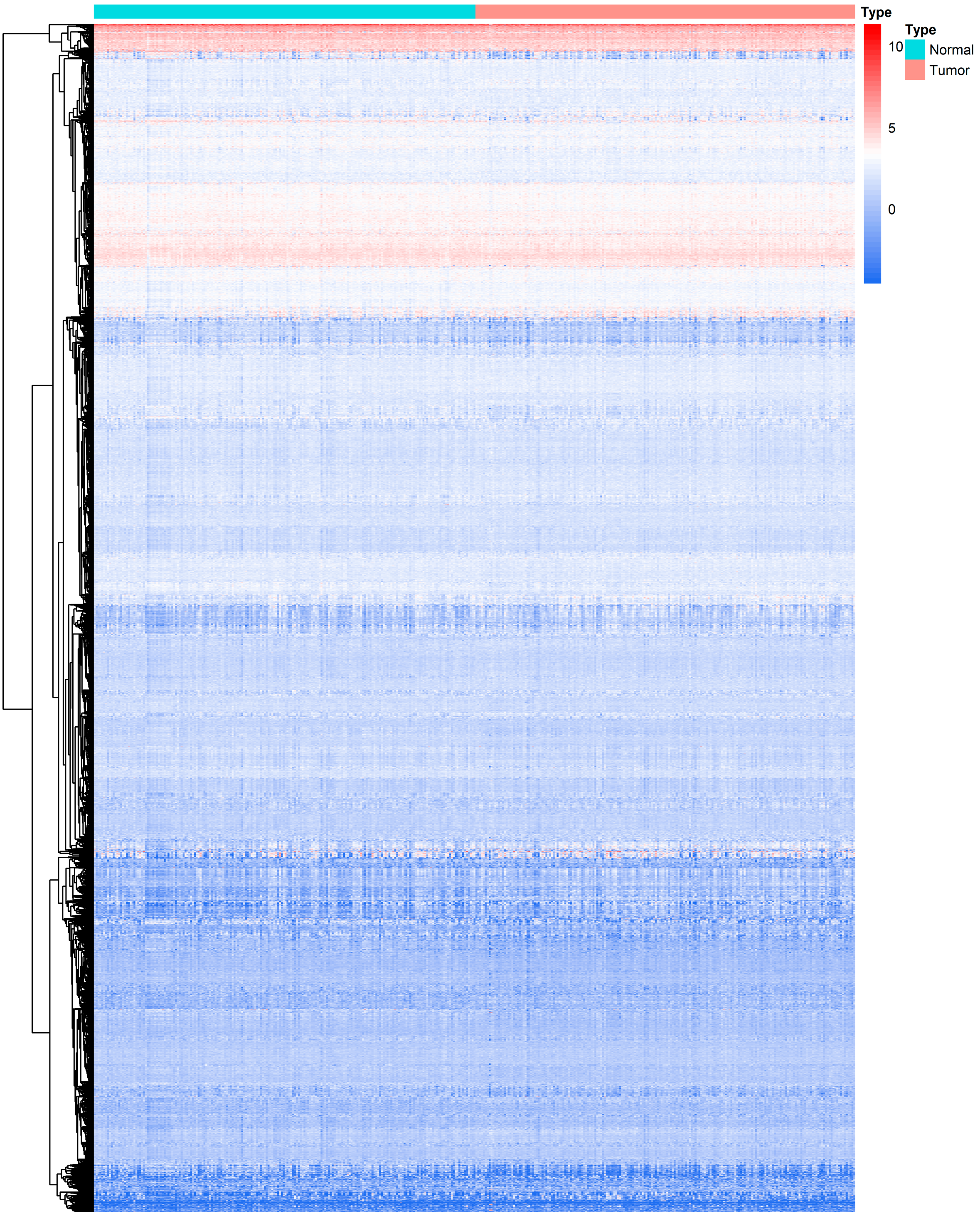

Figure S2. Analysis of differentially expressed genes between the low- and high-risk groups ($p < 0.001$, $|log2FC| > 1.0$).

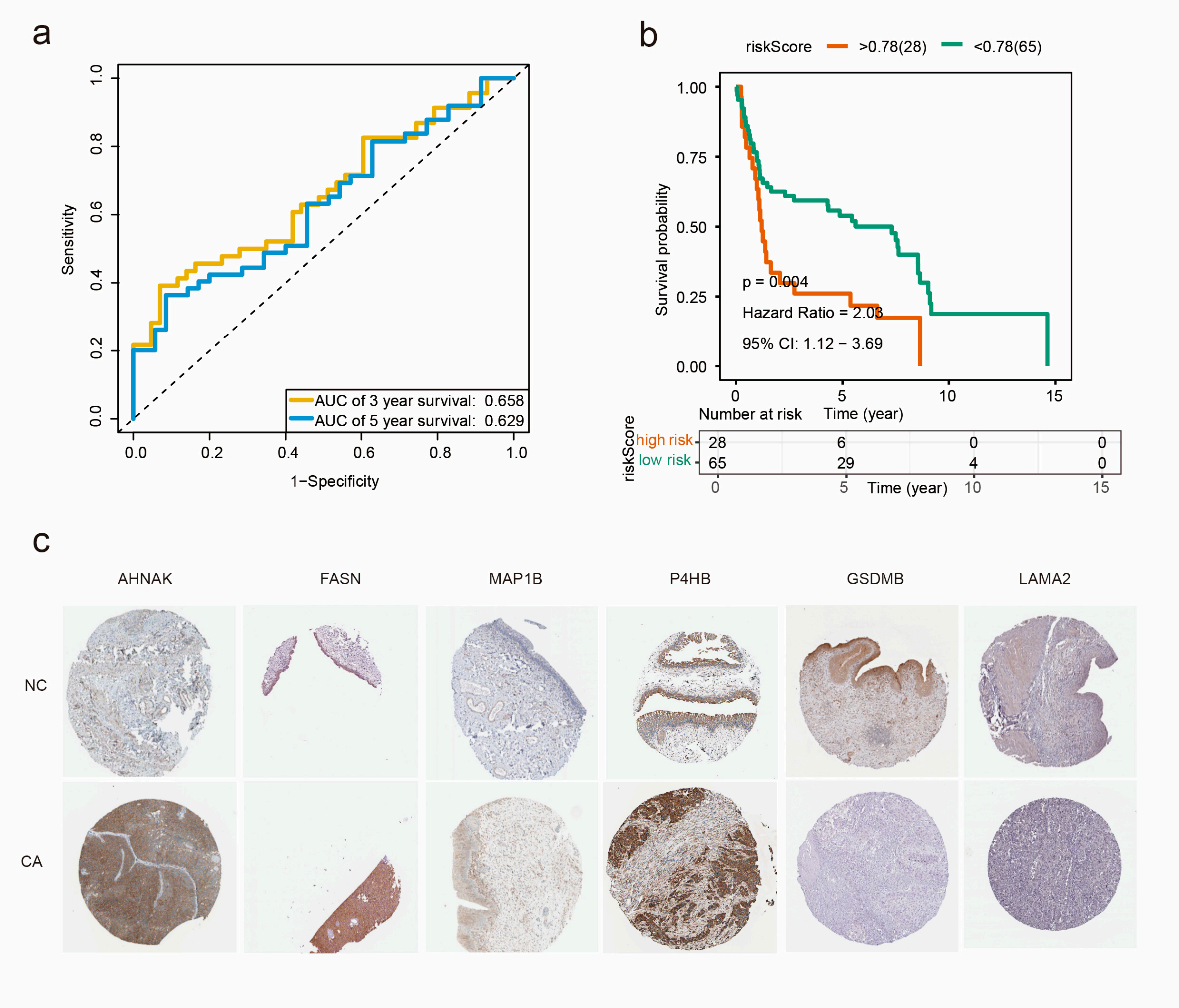

Figure S3. ROC curve for the test set (GSE31684); b The test set (GSE31684) survival analysis curves of the six RBPs were derived by creating the prognostic model shown in the forest map. Yellow indicates patients in the high-risk group, and blue indicates patients in the low-risk group; c Immunohistochemical results of the six RBPs in BC from the Human Protein Atlas databas.

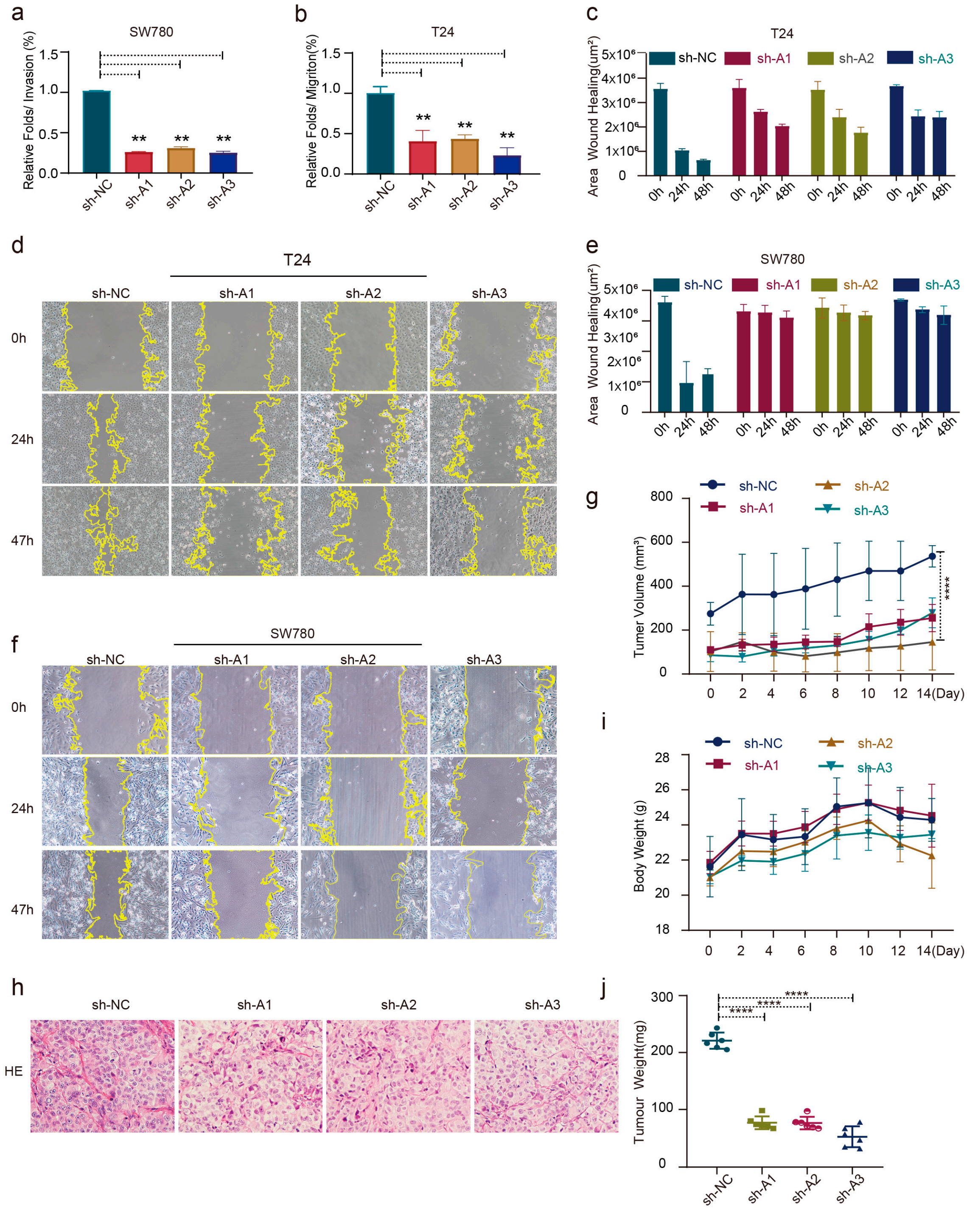

Figure S4. Analysis results of AHNAK-knockdown (KD) SW780 migration (a) and T24 invasion (b). AHNAK KD T24 and SW780 cells were observed using a microscope 0, 24, and 48 h after the scratch (c–f). Results of tumor volume (g) and weight analysis (i). H&E staining analysis of tumor tissues (h) (* $p < 0.05$, ** $p < 0.01$, *** $p < 0.001$, and **** $p < 0.0001$).

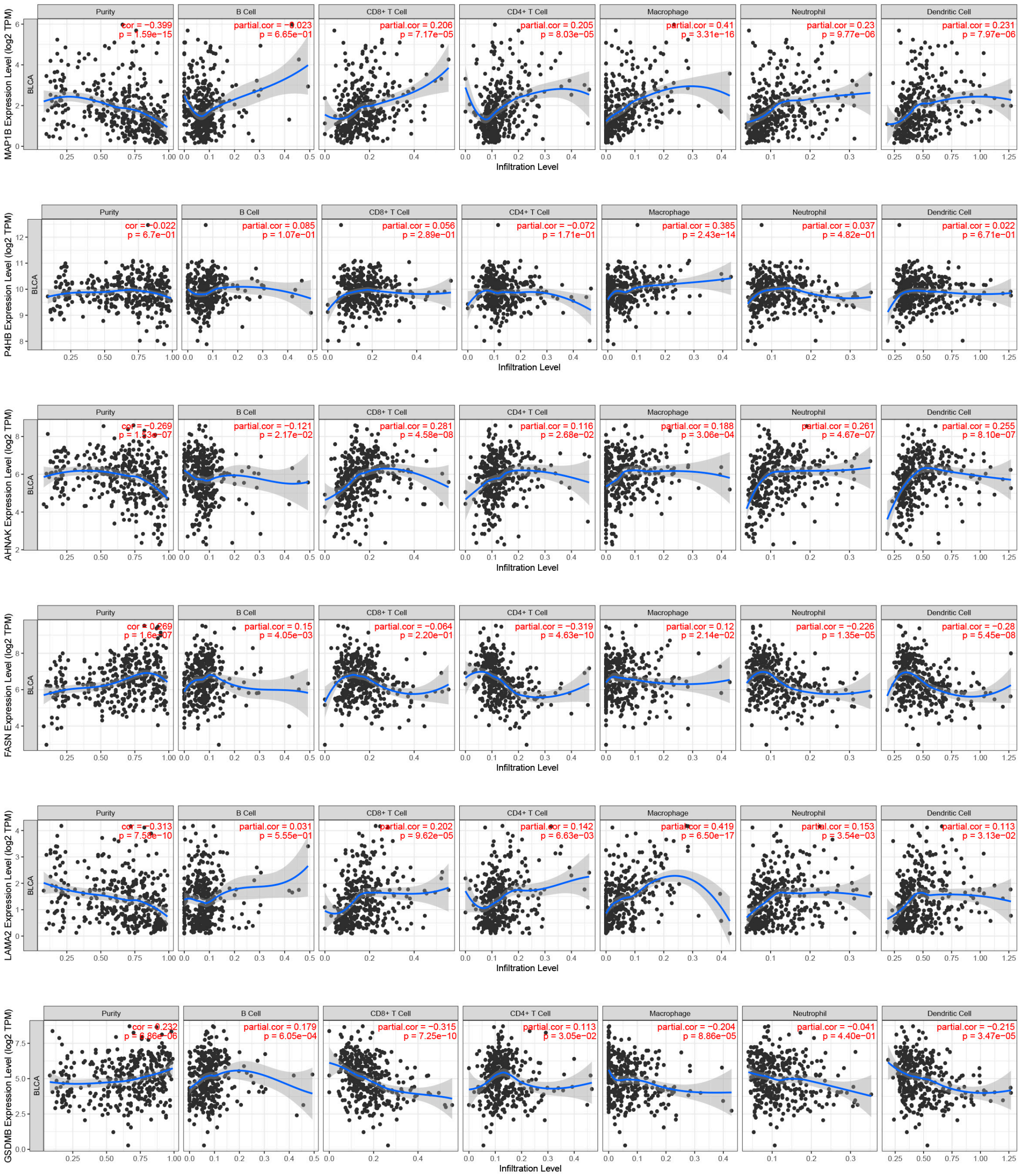

Figure S5. Correlation of six-RBP expression with macrophage- and neutrophil-immune infiltration levels in BC.
