# Supplementary figures and images for: Integrated Analysis and Identification of Critical RNA-Binding Proteins in Bladder Cancer
Source: Cancers (Basel). 2022 Jul 31;14(15):3739. doi: 10.3390/cancers14153739 (PMC9367304; doi:10.3390/cancers14153739)

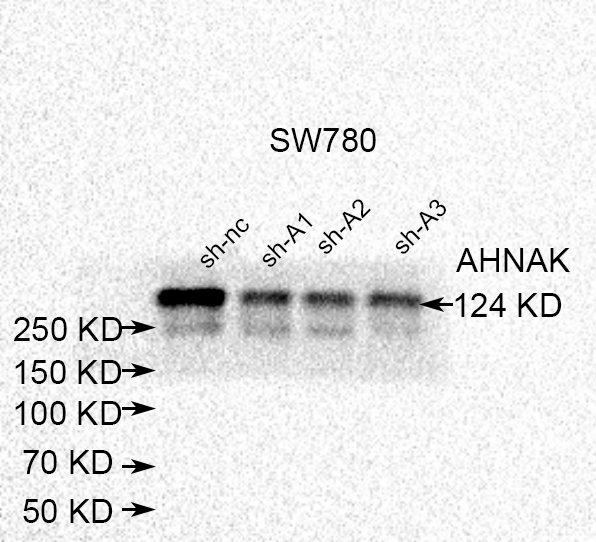

Supplement: Supplementary file 1 [file cancers-14-03739-s001.zip › WB-RAW DATA/AHNAK-KD/SW780-AHNAK-KD-2.tif]

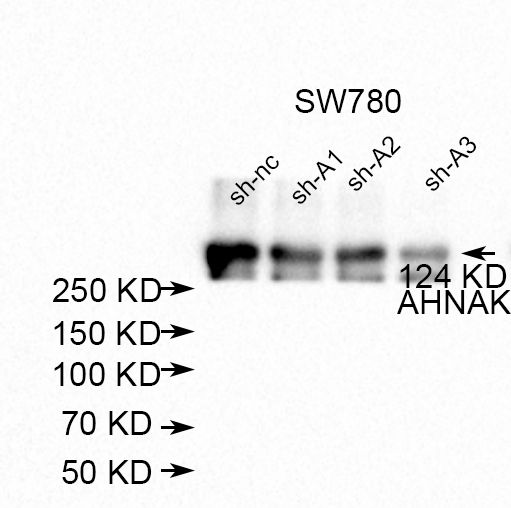

Supplement: Supplementary file 1 [file cancers-14-03739-s001.zip › WB-RAW DATA/AHNAK-KD/SW780-AHNAK-KD1.tif]

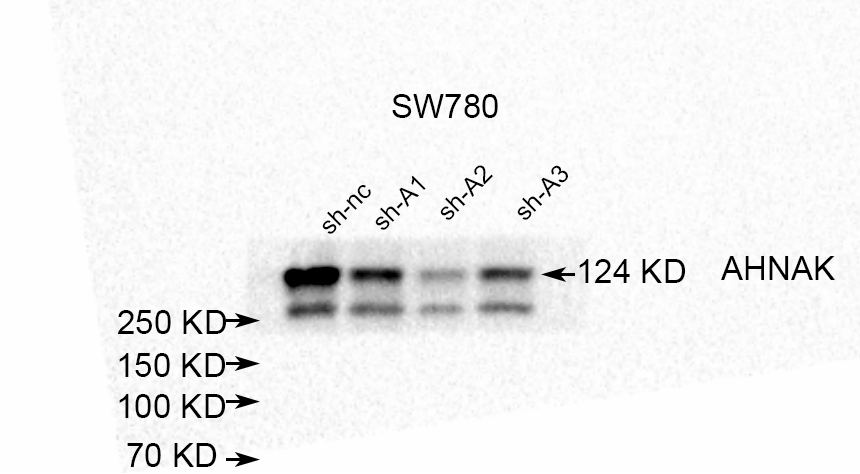

Supplement: Supplementary file 1 [file cancers-14-03739-s001.zip › WB-RAW DATA/AHNAK-KD/SW780-AHNAK-KD3.tif]

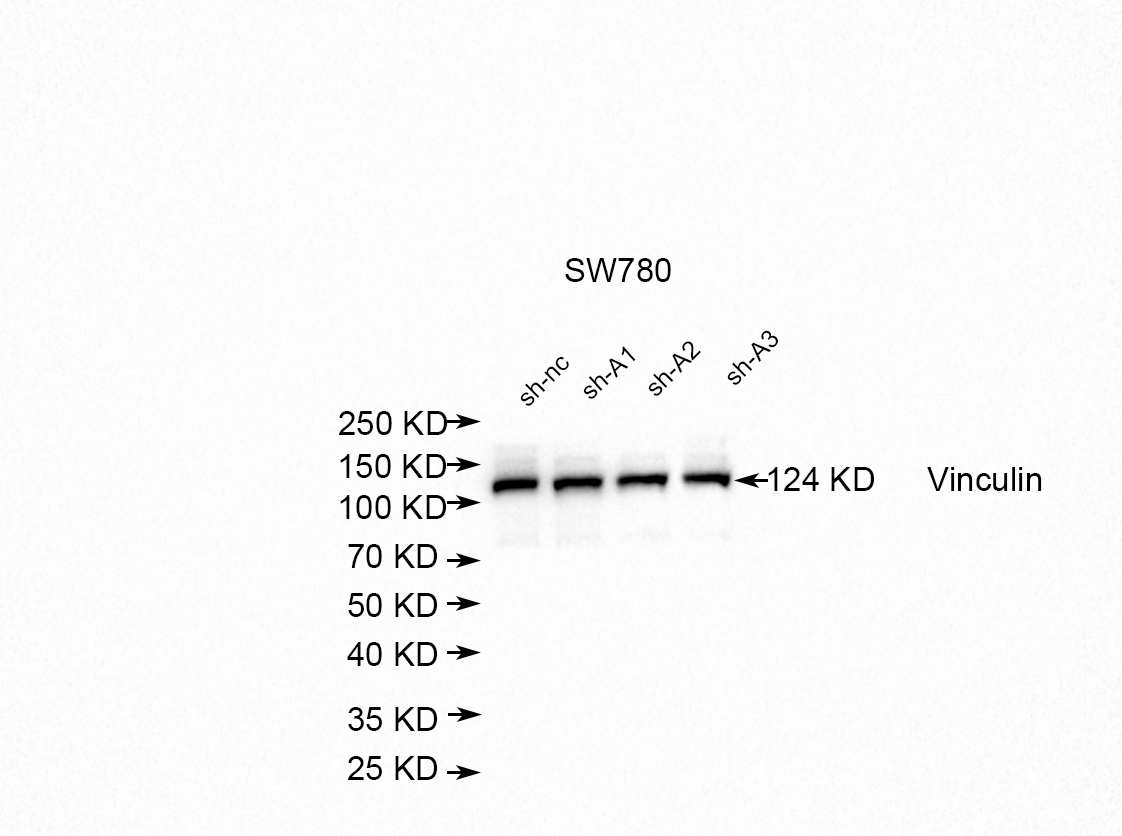

Supplement: Supplementary file 1 [file cancers-14-03739-s001.zip › WB-RAW DATA/AHNAK-KD/SW780-VICULIN-1.tif]

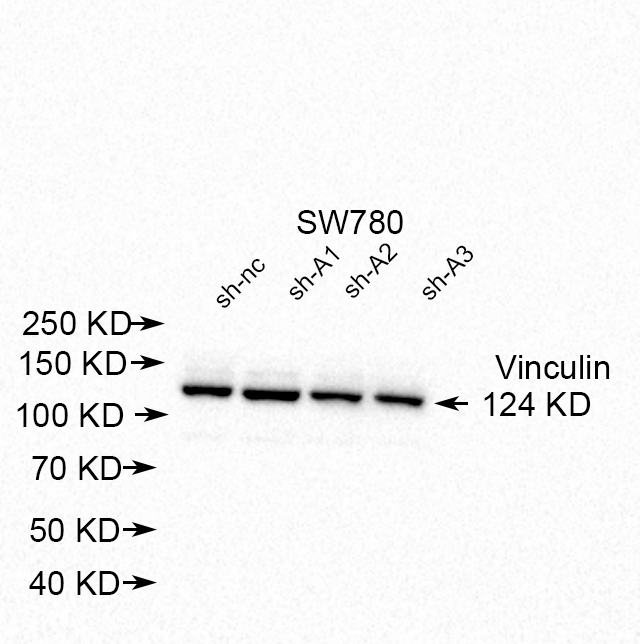

Supplement: Supplementary file 1 [file cancers-14-03739-s001.zip › WB-RAW DATA/AHNAK-KD/SW780-VICULIN-2.tif]

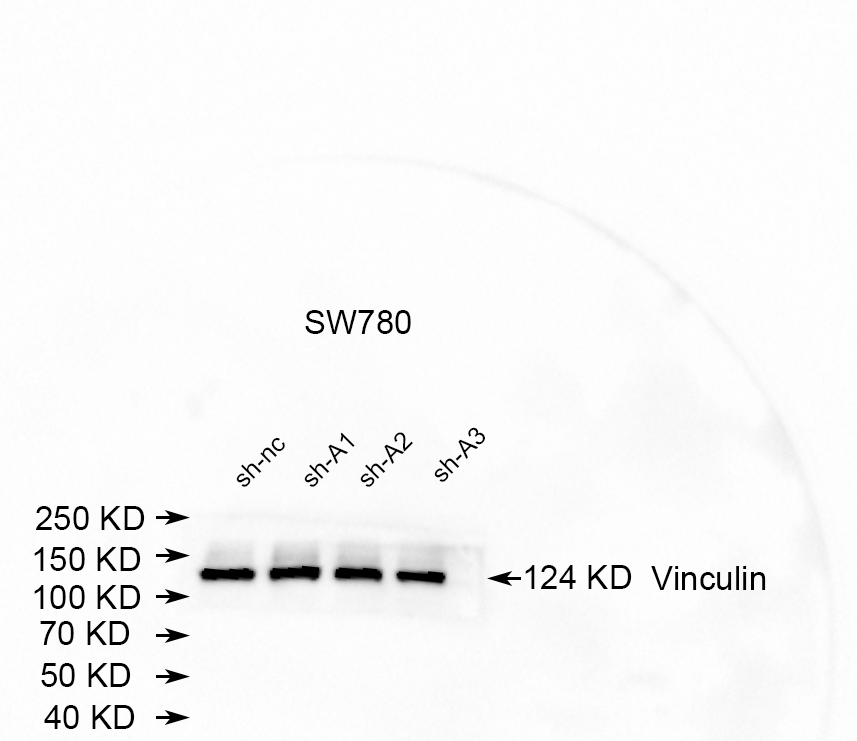

Supplement: Supplementary file 1 [file cancers-14-03739-s001.zip › WB-RAW DATA/AHNAK-KD/SW780-VICULIN-3.tif]

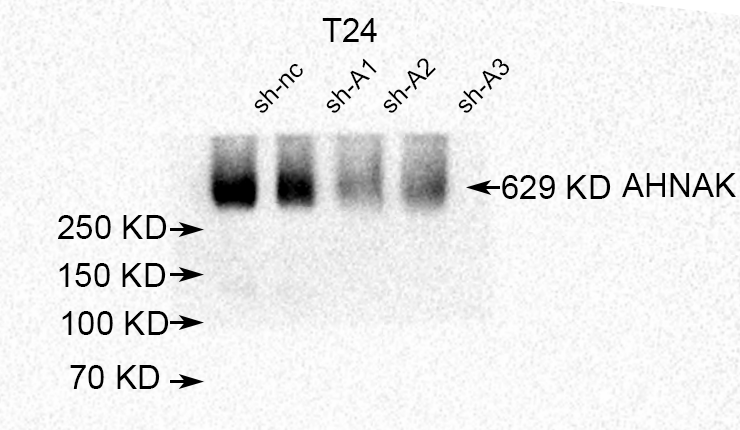

Supplement: Supplementary file 1 [file cancers-14-03739-s001.zip › WB-RAW DATA/AHNAK-KD/T24-AHNAK-KD-1.tif]

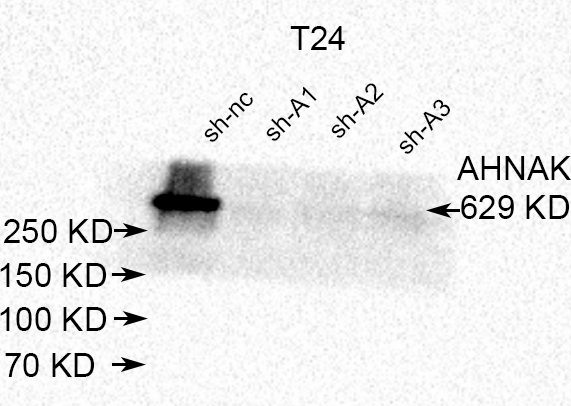

Supplement: Supplementary file 1 [file cancers-14-03739-s001.zip › WB-RAW DATA/AHNAK-KD/T24-AHNAK-KD2.tif]

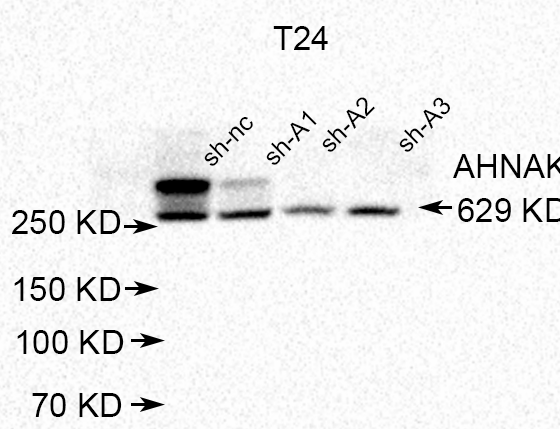

Supplement: Supplementary file 1 [file cancers-14-03739-s001.zip › WB-RAW DATA/AHNAK-KD/T24-AHNAK-KD3.tif]

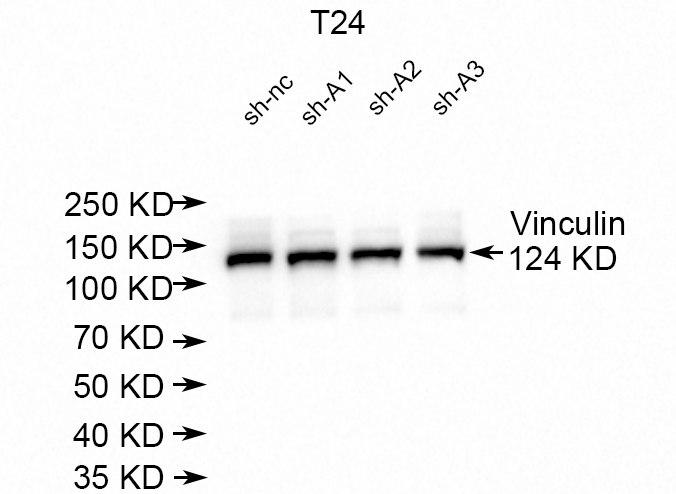

Supplement: Supplementary file 1 [file cancers-14-03739-s001.zip › WB-RAW DATA/AHNAK-KD/T24-VICULIN-2.tif]

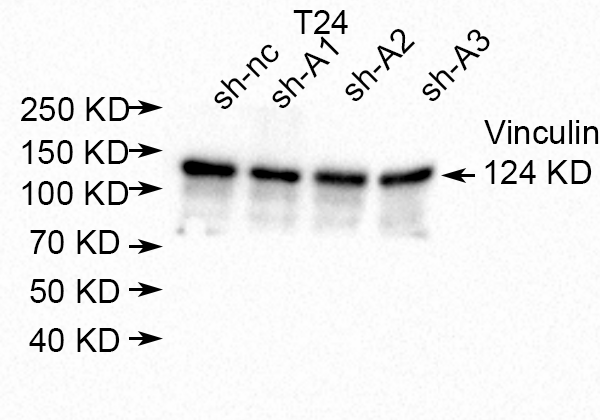

Supplement: Supplementary file 1 [file cancers-14-03739-s001.zip › WB-RAW DATA/AHNAK-KD/T24-VICULIN.tif]

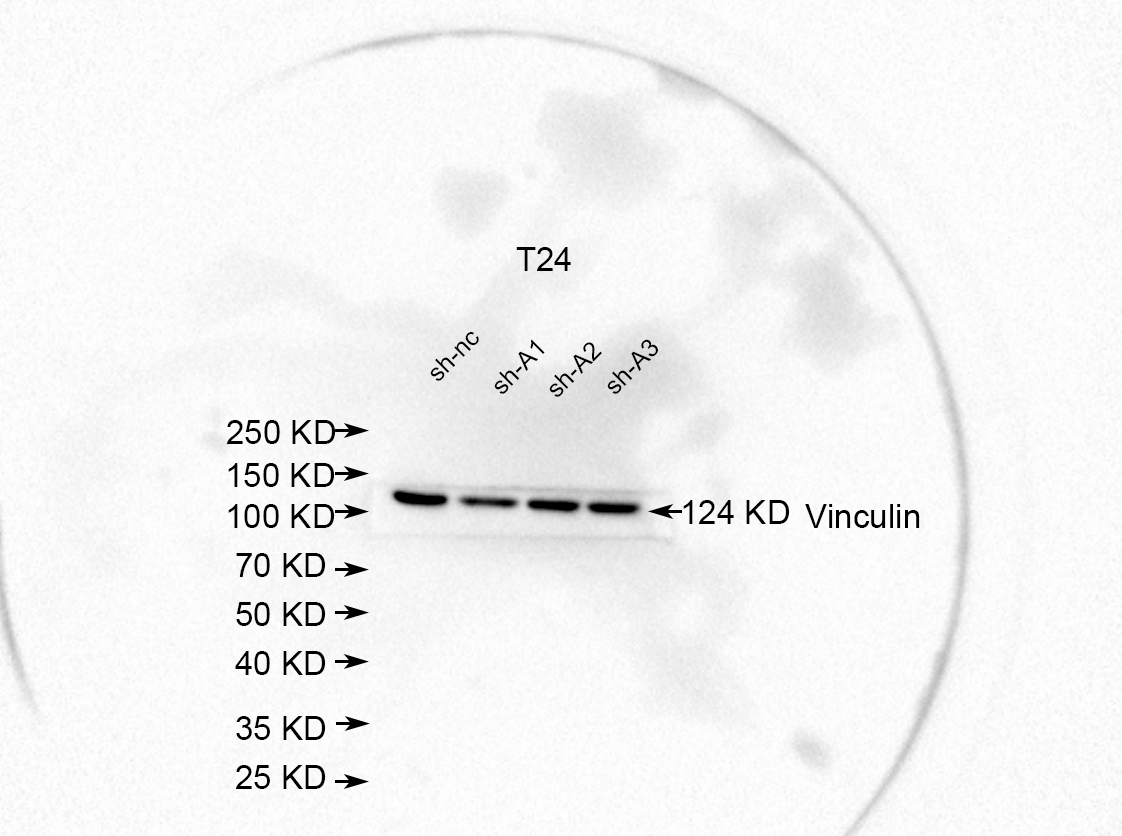

Supplement: Supplementary file 1 [file cancers-14-03739-s001.zip › WB-RAW DATA/AHNAK-KD/VICULIN-3.tif]

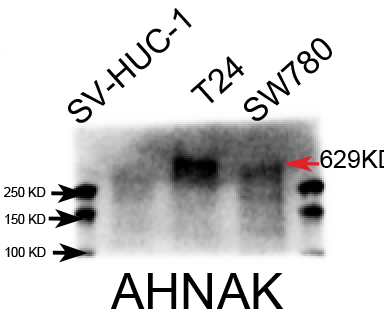

Supplement: Supplementary file 1 [file cancers-14-03739-s001.zip › WB-RAW DATA/WB-Expression of 6RBPS in cell lines/AHNAK-3.tif]

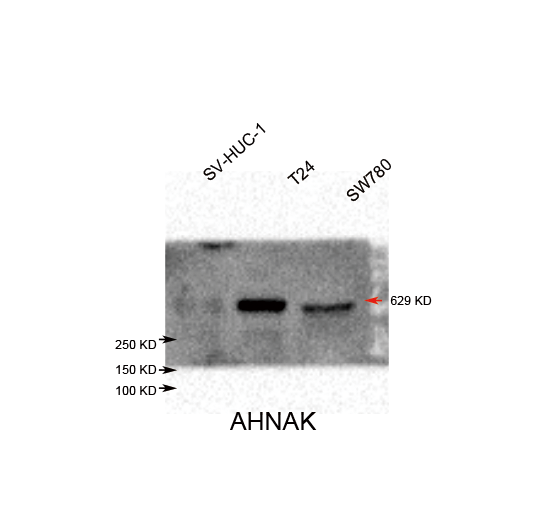

Supplement: Supplementary file 1 [file cancers-14-03739-s001.zip › WB-RAW DATA/WB-Expression of 6RBPS in cell lines/AHNAK1.tif]

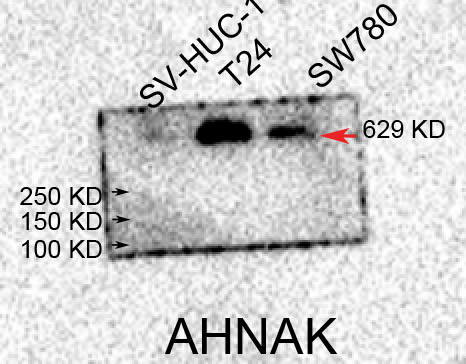

Supplement: Supplementary file 1 [file cancers-14-03739-s001.zip › WB-RAW DATA/WB-Expression of 6RBPS in cell lines/AHNAK2.tif]

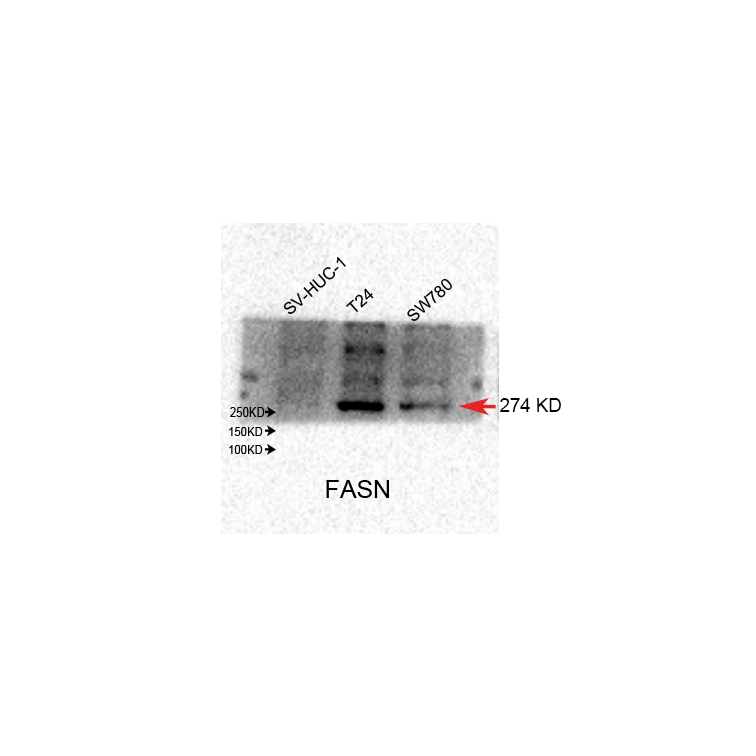

Supplement: Supplementary file 1 [file cancers-14-03739-s001.zip › WB-RAW DATA/WB-Expression of 6RBPS in cell lines/FASN1.tif]

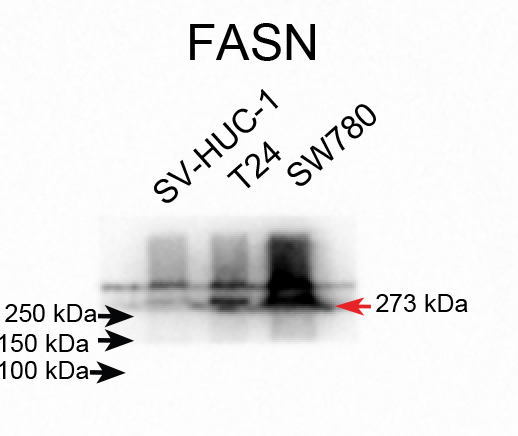

Supplement: Supplementary file 1 [file cancers-14-03739-s001.zip › WB-RAW DATA/WB-Expression of 6RBPS in cell lines/FASN3.tif]

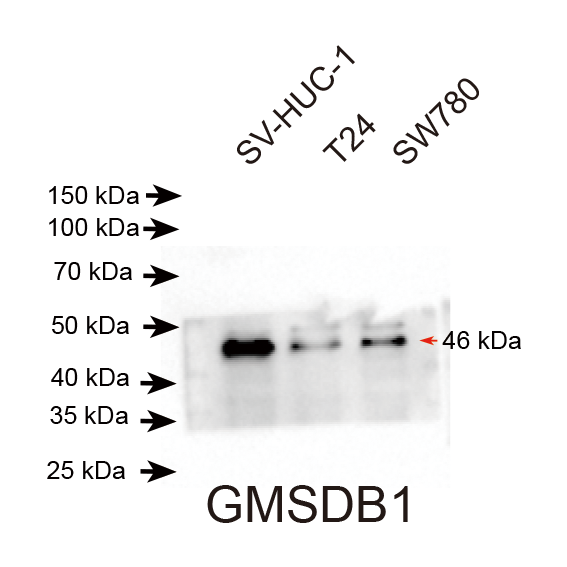

Supplement: Supplementary file 1 [file cancers-14-03739-s001.zip › WB-RAW DATA/WB-Expression of 6RBPS in cell lines/GMSDB.2.tif]

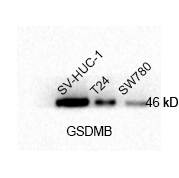

Supplement: Supplementary file 1 [file cancers-14-03739-s001.zip › WB-RAW DATA/WB-Expression of 6RBPS in cell lines/GMSDB1.tif]

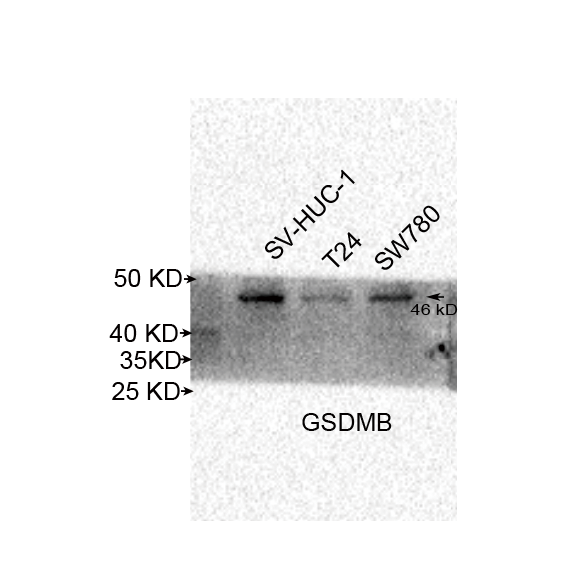

Supplement: Supplementary file 1 [file cancers-14-03739-s001.zip › WB-RAW DATA/WB-Expression of 6RBPS in cell lines/GMSDB2.tif]

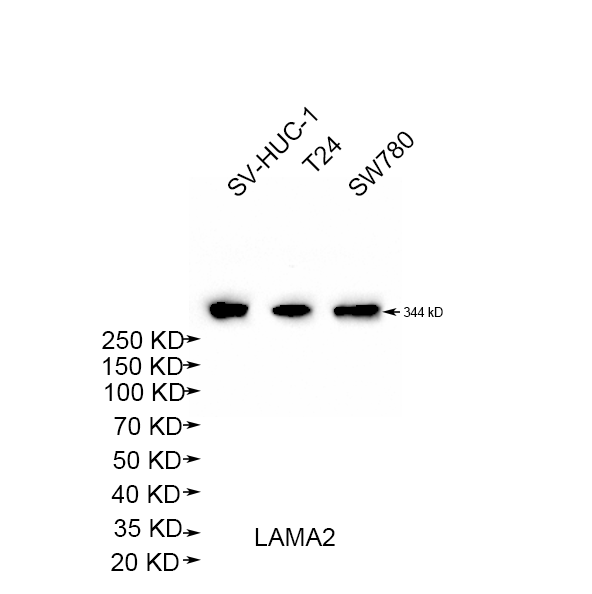

Supplement: Supplementary file 1 [file cancers-14-03739-s001.zip › WB-RAW DATA/WB-Expression of 6RBPS in cell lines/LAMA2.1.tif]

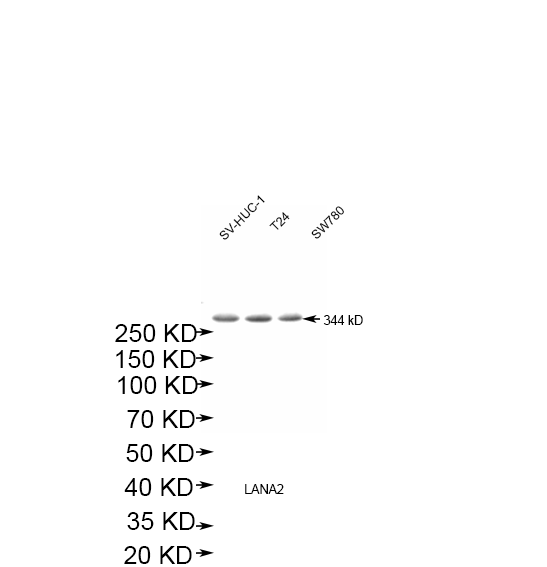

Supplement: Supplementary file 1 [file cancers-14-03739-s001.zip › WB-RAW DATA/WB-Expression of 6RBPS in cell lines/LAMA2.2.tif]

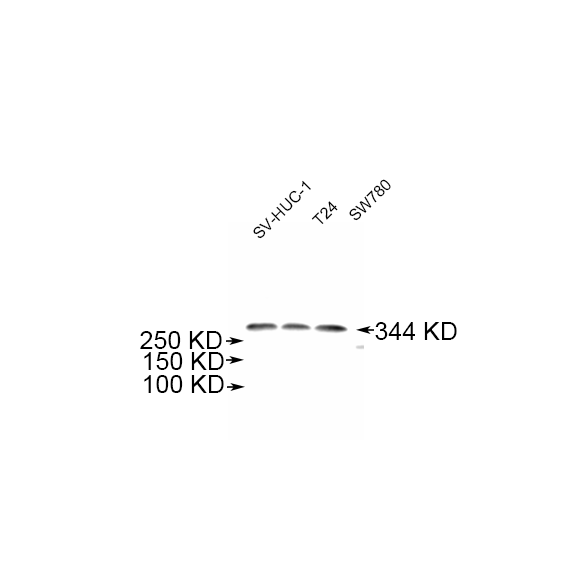

Supplement: Supplementary file 1 [file cancers-14-03739-s001.zip › WB-RAW DATA/WB-Expression of 6RBPS in cell lines/LAMA2.3.tif]

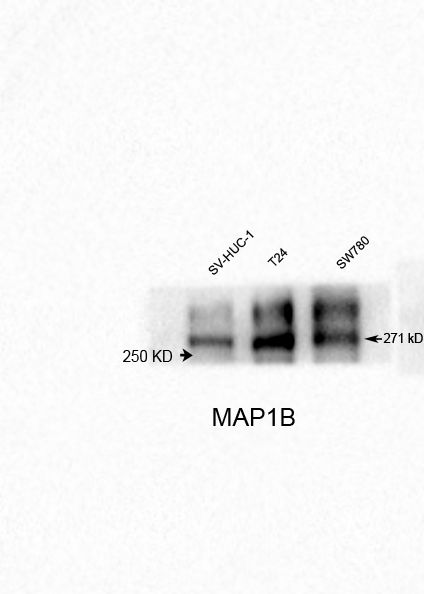

Supplement: Supplementary file 1 [file cancers-14-03739-s001.zip › WB-RAW DATA/WB-Expression of 6RBPS in cell lines/MAP1B.1.tif]

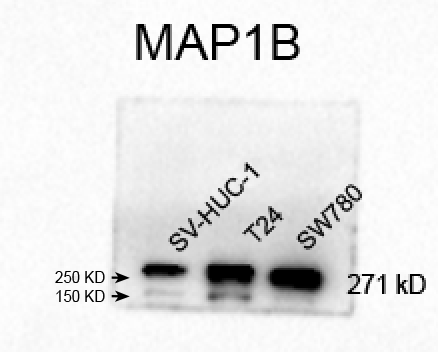

Supplement: Supplementary file 1 [file cancers-14-03739-s001.zip › WB-RAW DATA/WB-Expression of 6RBPS in cell lines/MAP1B.3.tif]

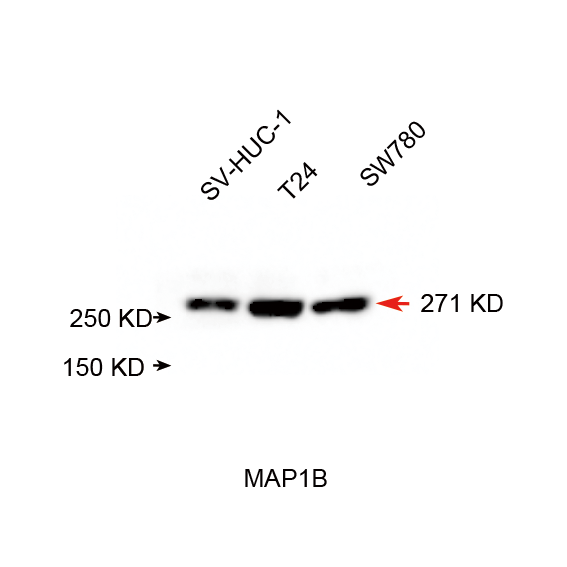

Supplement: Supplementary file 1 [file cancers-14-03739-s001.zip › WB-RAW DATA/WB-Expression of 6RBPS in cell lines/MAP1B.tif]

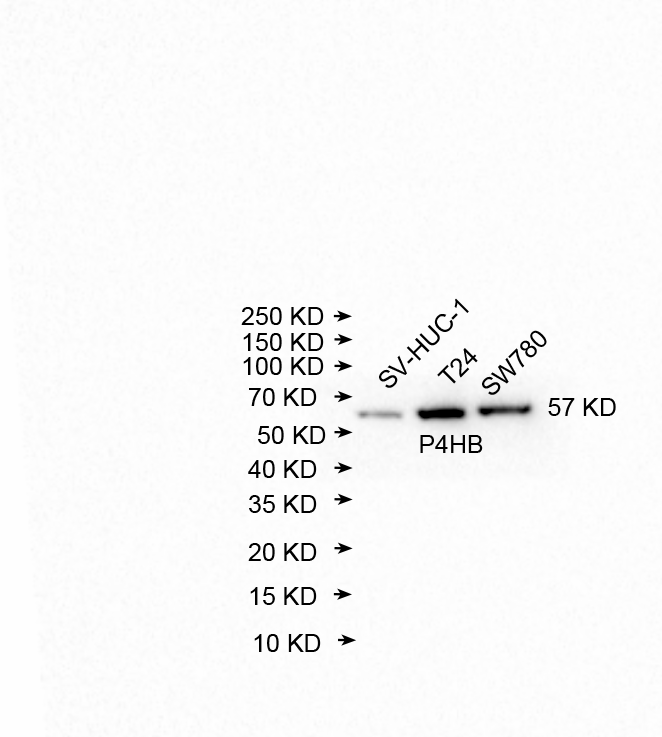

Supplement: Supplementary file 1 [file cancers-14-03739-s001.zip › WB-RAW DATA/WB-Expression of 6RBPS in cell lines/P4HB.1.tif]

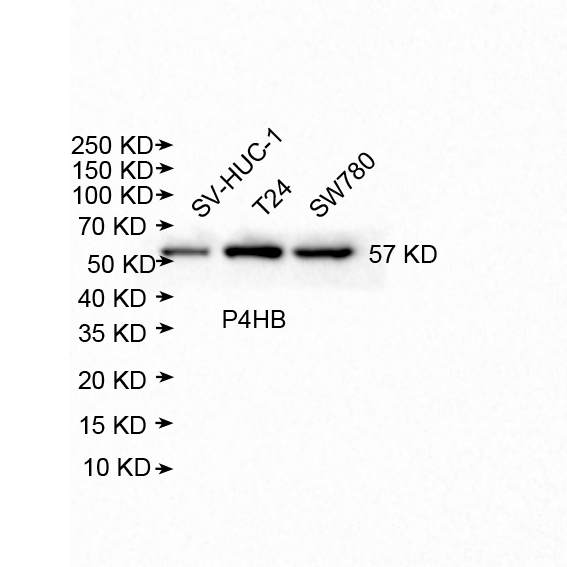

Supplement: Supplementary file 1 [file cancers-14-03739-s001.zip › WB-RAW DATA/WB-Expression of 6RBPS in cell lines/P4HB.2.tif]

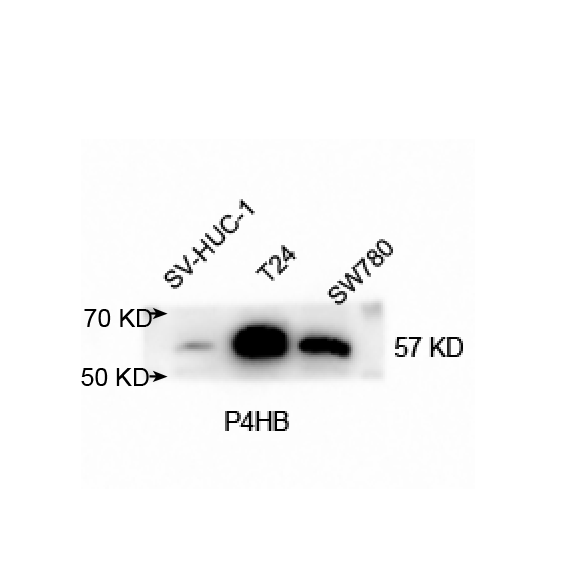

Supplement: Supplementary file 1 [file cancers-14-03739-s001.zip › WB-RAW DATA/WB-Expression of 6RBPS in cell lines/P4HB.3.tif]

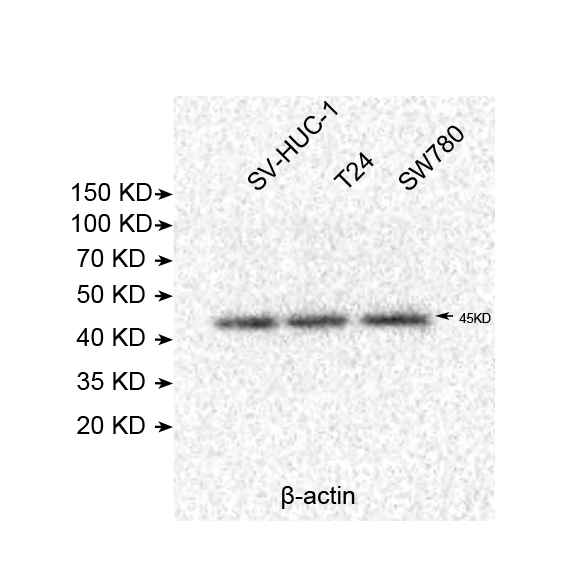

Supplement: Supplementary file 1 [file cancers-14-03739-s001.zip › WB-RAW DATA/WB-Expression of 6RBPS in cell lines/a┬-actin.tif]

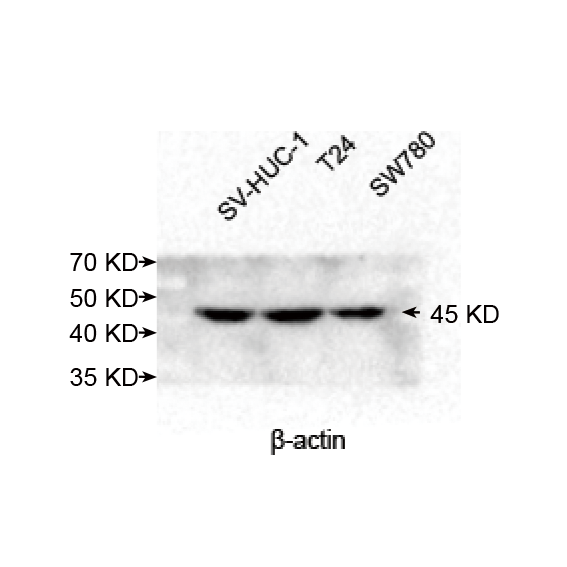

Supplement: Supplementary file 1 [file cancers-14-03739-s001.zip › WB-RAW DATA/WB-Expression of 6RBPS in cell lines/a┬-actin1.tif]

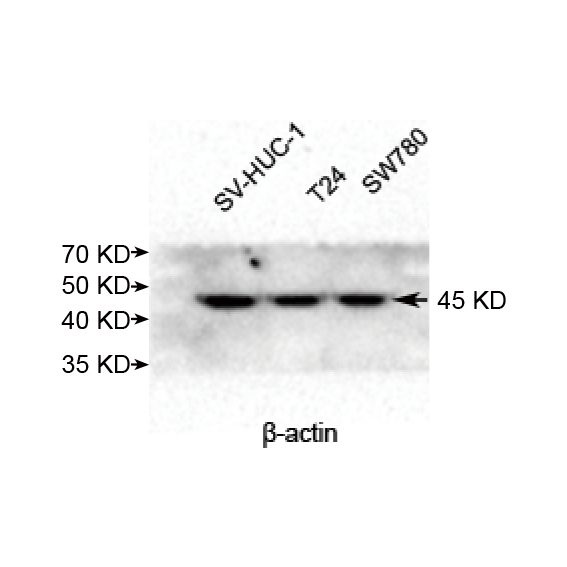

Supplement: Supplementary file 1 [file cancers-14-03739-s001.zip › WB-RAW DATA/WB-Expression of 6RBPS in cell lines/a┬-actin3.tif]
